# Computer-Aided Screening and Revealing Action Mechanism of Green Tea Polyphenols Intervention in Alzheimer’s Disease

**DOI:** 10.3390/foods12030635

**Published:** 2023-02-02

**Authors:** Min Wang, Xiaotang Yang, Yilin Gao, Weiwei Han

**Affiliations:** Key Laboratory for Molecular Enzymology and Engineering of Ministry of Education, School of Life Science, Jilin University, 2699 Qianjin Street, Changchun 130012, China

**Keywords:** tea polyphenols, Alzheimer’s disease, network pharmacology, molecular docking, quantum chemical calculation

## Abstract

The accumulation of cross-β-sheet amyloid fibrils is a hallmark of the neurodegenerative process of Alzheimer’s disease (AD). Although it has been reported that green tea substances such as epicatechin (EC), epicatechin-3-gallate (ECG), epigallocatechin (EGC) and epigallocatechin-3-gallate (EGCG) could alleviate the symptoms of AD and other neurodegenerative diseases, the pharmacological mechanism remains largely unexplored. This study aimed to reveal the underlying mechanism of EC, ECG, EGC and EGCG in AD using a computer-aided screening strategy. Our results showed that the four tea polyphenols interfered with the signaling pathways of AD via calcium signaling channels, neurodegeneration-multiple disease signal pathways and others. We also identified the key residues of the interaction between VEGFA and the four active components, which included Glu64 and Phe36. Overall, we have provided valuable insights into the molecular mechanism of tea polyphenols, which could be used as a reference to improve therapeutic strategies against AD.

## 1. Introduction

Alzheimer’s disease (AD) is a prevalent neurodegenerative disorder that significantly affects the daily activities and quality of life of the elderly population [1,2,3,4,5,6,7,8]. Its etiology has been reported to be multifactorial, including family history and genetics, advanced age, head trauma, cognitive impairment, etc. [1,2,3,4,5,6,7,8]. Currently, there are several hypotheses describing the pathogenesis of AD, such as β-amyloid (Aβ) deposition [9,10,11,12] and tau hyperphosphorylation [13], which are reported to cause extracellular amyloid plaques and neurofibrillary tangle in brain neurons [14]. Despite advancements in pharmacology, the incidence of AD has been increasing as there is neither a cure nor treatment to alter its progression, and existing anti-AD drugs are clinically limited due to having poor efficacy and causing significant adverse reactions [15,16].

The health benefits of tea have been recognized worldwide [17,18,19]. Most epidemiological studies suggest that drinking two to three cups of tea daily, particularly green or black tea, could be a natural complementary therapy for neurodegenerative diseases as it can reduce the risk of cognitive impairment [20]. In addition, there is abundant literature on the neuroprotective effects of tea, such as improved learning and memory, reduced risk of toxicity and good synergy with anti-AD medications [21,22,23].

The regulating effects of the active ingredients in tea on AD can be divided into direct and indirect effects. The active ingredients of tea can directly exert their effects on the nervous system and the immune system following intestinal absorption. For instance, tea polyphenols, in particular, epicatechin (EC) [24], epicatechin-3-gallate (ECG) [25], epigallocatechin (EGC) [26] and epigallocatechin- 3-gallate (EGCG) [27], have been extensively reported to offer protection against neurodegenerative diseases such as AD and Parkinson’s disease [28,29,30,31]. Most existing related studies have focused on these polyphenols’ antioxidant and anti-inflammatory properties [24,25,26,27]. Furthermore, EGCG, the major constituent of green tea, has been confirmed to inhibit Aβ fibril formation [32]. However, the underlying mechanism of the inhibition of Aβ fibril formation by these polyphenols has been seldom reported. 

Network pharmacology is an interactive network based on the concept of “disease-gene-target-compounds” that can be used to assess the potential effects of tea polyphenols (especially EC [24], ECG [25], EGC [26] and EGCG [27]) in patients with AD from a systematic and comprehensive perspective to reveal the complex underlying mechanisms of these active compounds [33,34,35]. On the basis of this concept, we performed a computer-aided screening strategy to explore the mechanism and molecular targets of tea polyphenol interventions in AD. In addition, the interaction mechanisms between hub targets and tea polyphenols were assessed via molecular docking. Overall, this research aimed to provide some insights into the precise target information for preventing and alleviating AD using tea polyphenols.

## 2. Materials and Methods

### 2.1. Prediction of AD-Related Targets and Tea Polyphenols Targets 

First, we screened for potential AD target genes from the DisGeNET (https://www.disgenet.org/, accessed on 25 October 2022) [36], GeneCards (https://www.genecards.org/, accessed on 25 October 2022) [37], PharmGKB (https://www.pharmgkb.org/, accessed on 24 October 2022) [38] and UniProt (https://www.uniprot.org/, accessed on 25 October 2022) [39] databases. In total, we identified 220 potential AD target genes with a score > 0.1 in DisGeNET, 1300 potential targets with a score > 1 in GeneCards, and 20 potential targets in PharmGKB. Second, after the union of the three databases was deleted, 1407 potential AD target genes remained. Then, we used the SEA (http://sea.bkslab.org/, accessed on 20 November 2022) [40] and Super-Pred [41] (https://prediction.charite.de/subpages/target_prediction.php, accessed on 20 November 2022) databases for predicting the target genes of EC, ECG, EGC and EGCG based on the intersection of active components and disease target genes, which yielded 46, 12, 43 and 57 related intersection genes, respectively.

### 2.2. Construction of the Protein–Protein Interaction (PPI) Network

The PPI network was constructed using the STRING database (http://string-db.org/, accessed on 22 November 2022) [42] to assess the potential interactions between the screened hub targets. The interactions between hub targets were subsequently displayed using Cytoscape software (version 3.9.1; https://cytoscape.org/, accessed on 22 November 2022) [43]. Then, the topological characteristics of the PPI networks were analyzed, and the top 10 hub genes were selected using the analytical tool of Cytoscape. 

### 2.3. GO and KEGG Enrichment Analysis

R packages such as BiocManager, clusterProfiler, AnnotationHub, org.Hs.eg, pathview, dplyr and ggplot2 were used for enrichment analysis of Gene Ontology (GO) [44,45] biological processes and Kyoto Encyclopedia of Genes and Genomes (KEGG) according to the core target information. Using a cut-off of *p* = 0.01 and q = 0.01, we retrieved GO information from org.Hs.eg.Db of Bioconductor. The final results were visualized using bar charts and bubble charts. 

### 2.4. Quantum Chemical Calculation

The quantitative calculation of the four tea polyphenols was performed by Gaussian09W and GaussView 5.0 [46]. The Method of Gaussian Calculation was B3LYP of DFT in Ground State, and we made the Basis Set 6–31G. Visualizations of the quantitative calculations were mapped by Multiwfn [47] and VMD [48], including HOMO-LUMO orbits, electrostatic potential (ESP), average local ionization energy (ALIE) and local electron affinity (LEA).

### 2.5. Complex Fingerprint Analysis

Discovery Studio was used to evaluate the interaction between the four tea polyphenols and the hub targets. On the basis of the results of the PPI analysis (intersection of the top 10 hub targets with high degree values), the crystal structure of VEGFA (4qaf, resolution: 1.80 Å) was chosen as the receptor protein from the RCSB protein data bank (http://www.rcsb.org/pdb, accessed on 24 November 2022) [49] for further molecular docking. The filtering criteria for selecting reporter proteins were organism “Homo” and “Resolution < 2.0”. Before docking, ligands and water molecules in the crystal structure of VEGFA were removed to prepare the VEGFA protein. Since there was no crystal structure for VEGFA with active small molecules, the active sites of VEGFA binding with peptides were selected to define the molecular docking sites. Then, the four kinds of tea polyphenols were used as ligand docking. Before docking, the energy of all small molecules was minimized by preparing small molecules and adding a CHARMm force field [50]. Additionally, batch docking was performed between the four tea polyphenols and VEGFA using LibDock [51]. The hydrogen bonds and coordinating interactions between the residues of receptor protein’s active sites and the tea polyphenols’ poses were identified using Discovery Studio [52] to conduct complex fingerprint analysis. Lastly, Pymol software [53] was used to show the details of the interaction between the tea polyphenols targets.

### 2.6. Alanine Scanning

The protein structures of tea polyphenols docking with VEGFA were imported into Discovery Studio and CHARMm force field [50]. We selected 3 Å amino acid residues around the ligands (four tea polyphenol small molecules) for single-point alanine mutation, and the binding energy after the mutation was calculated to screen key residues.

## 3. Results

**Figure 1:**
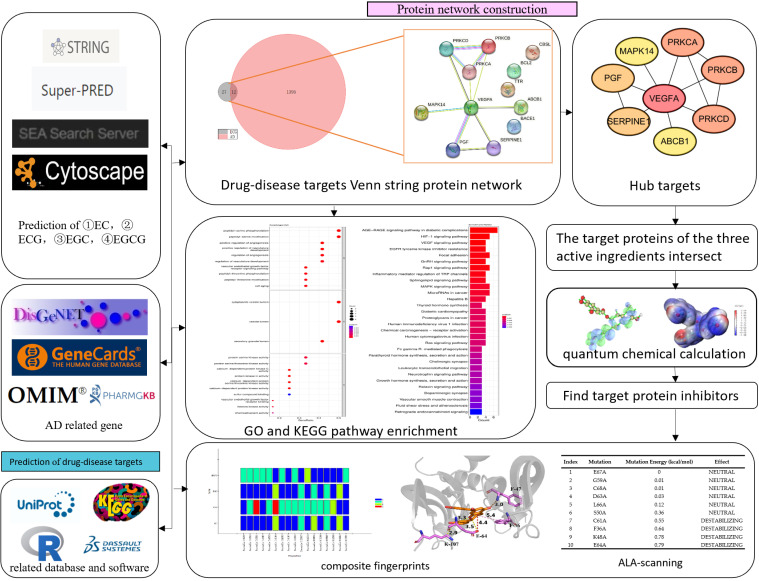
Workflow of the network pharmacology methods demonstrated the core targets and molecular mechanism related to the activity of tea polyphenols on treating AD. It contained the databases screening, PPI network mapping, target gene identification, GO and KEGG pathway enrichment analysis, quantum chemical calculation, and molecular docking.

Taking ECG as an example, the workflow for investigating the anti-AD-related mechanism of tea polyphenols using network pharmacological methods is shown in Figure 1. First, we collected and compared the retrieved gene data between tea polyphenols and AD from different online databases such as Super-Pred [41], DisGeNET [36], GeneCards [37], and others. Then, based on the cross-genes data, bioinformatics analysis was conducted to reveal the potential significance of tea polyphenols on AD, including underlying biological processes, pathways and mechanisms. Quantitative calculation and analysis of the four tea polyphenol small molecules were performed to explore their characteristics using Gaussian 09. Moreover, Discovery Studio was used to perform molecular docking and alanine scanning between the main targeting protein (VEGFA) and the four active components.

### 3.1. Potential Targets of AD and Tea Polyphenols

**Figure 2:**
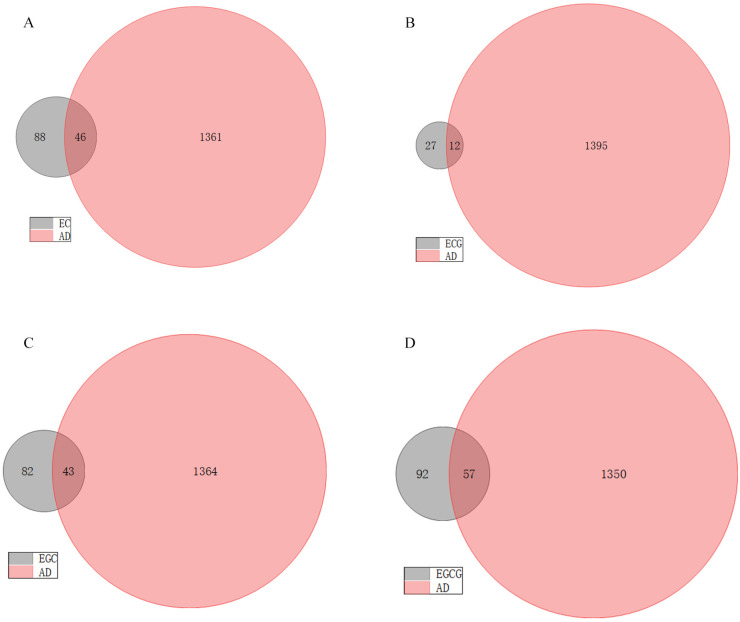
Venn diagrams showing the cross-genes shared between tea polyphenols and AD. (**A**) The number of common genes shared between EC and AD is 46. (**B**) The number of common genes shared between ECG and AD is 12. (**C**) The number of common genes shared between EGC and AD is 43. (**D**) The number of common genes shared between EGCG and AD is 57.

**Figure 3:**
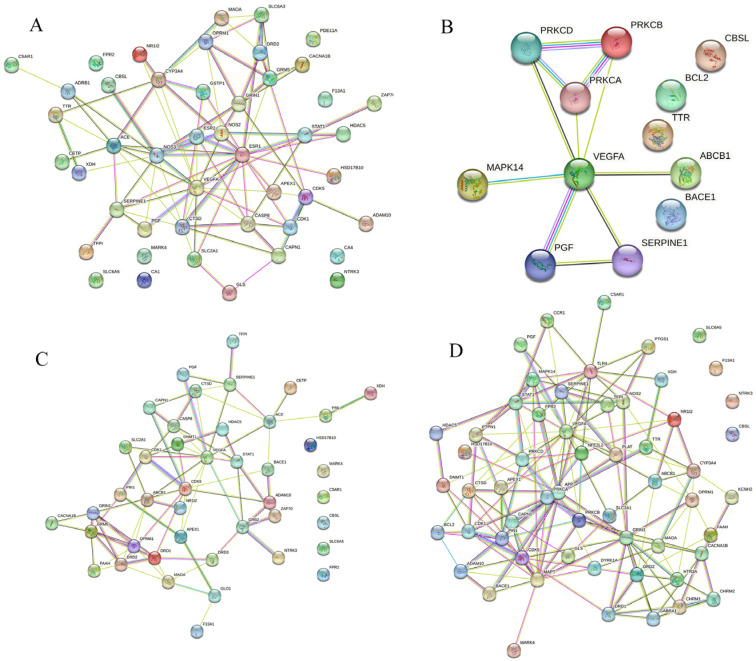
PPI network analysis using STRING database demonstrating the interaction of the shared genes found in tea polyphenols and AD. (**A**) PPI network of EC. (**B**) PPI network of ECG. (**C**) PPI network of EGC. (**D**) PPI network of EGCG.

We collected 1407 pathogenic genes/targets related to AD from the DisGeNET, GeneCards and PharmGKB databases. In addition, regarding the 134, 39, 125 and 149 pharmacological genes/targets of EC, ECG, EGC and EGCG that were initially detected, respectively, Venn diagrams revealed that 46, 12, 43 and 57 genes/targets of these respective tea polyphenols were significantly associated with AD (Figure 2). The PPI networks, including the shared targets, are mapped in Figure 3.

### 3.2. Hub Targets Collection of Tea Polyphenols Anti-AD

**Figure 4:**
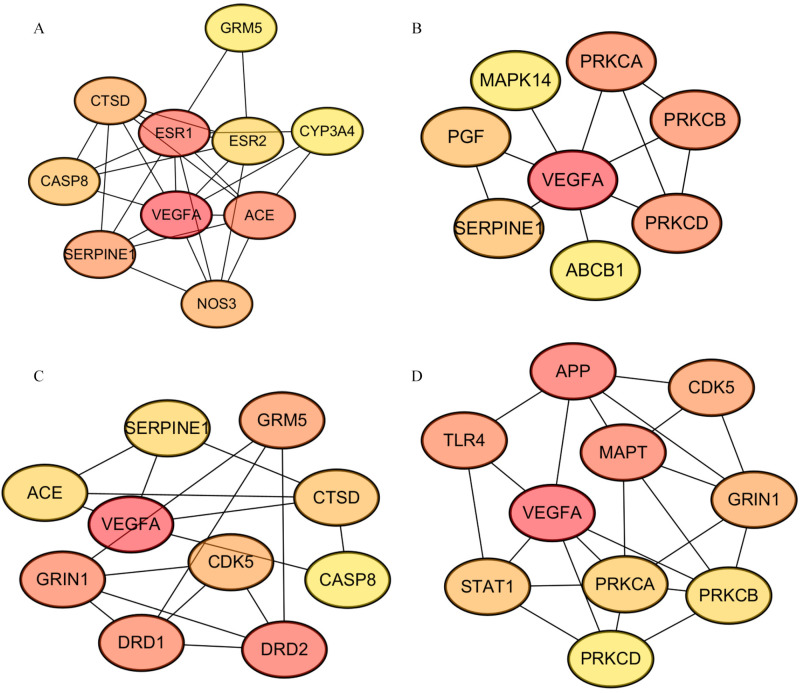
Protein interaction involved in the anti-AD activity of tea polyphenols. (**A**) The interaction of core targets of EC. (**B**) The interaction of core targets of ECG. (**C**) The interaction of core targets of EGC. (**D**) The interaction of core targets EGCG. Using cytoHubba, the interaction of hub targets related to the activity of tea polyphenols on treating AD was shown. By intersecting top10 hub genes, we understood that the key protein was VEGFA.

Using cytoHubba, a functionally relevant protein network of the tea polyphenol–AD system was created by applying a topological analysis method from Maximal Clique Centrality (MCC) [54]. Scores for estimating the relationship between nodes and edges were obtained using the MCC algorithm, whereby a higher score and darker color indicated a more significant correlation of the gene with AD. Then, we screened the top 10 target genes/proteins with the highest scores for each active ingredient. The important proteins detected included GRM5, CYP3A4, ACE, NOS3, SERPINE1, CASP8, CTSD, ESR1, ESR2, VEGFA, PRKCA, PRKCB, PRKCD, ABCB1, PGF, MAPK14, CASP8, DRD2, DRD1, GRIN1, CDK5, APP, STAT1, TLR4 and MAPT (Figure 4).

### 3.3. GO and KEGG Pathway Enrichment Analysis

**Figure 5:**
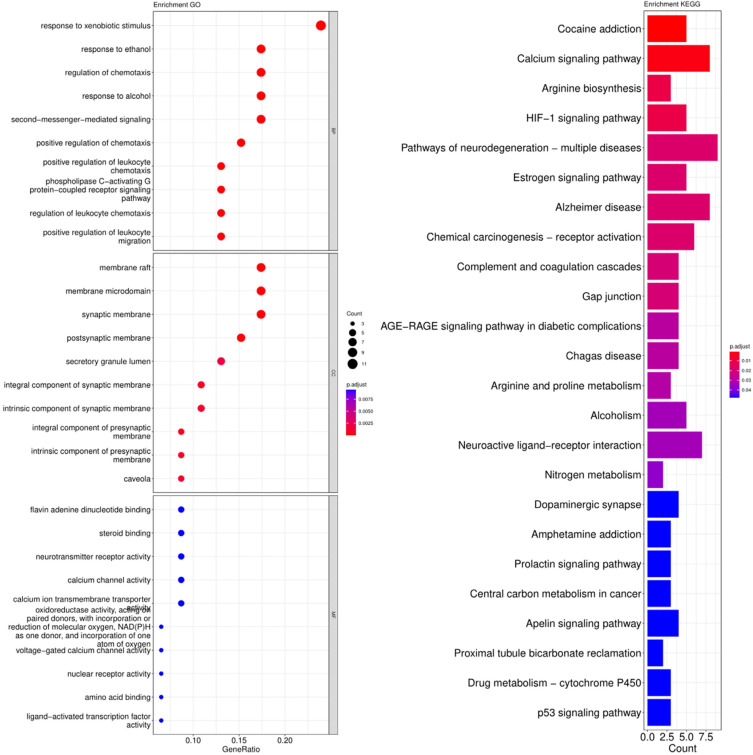
Results of GO and KEGG pathway enrichment analysis based on the common genes between EC and AD. The visualization of GO analysis related to EC against AD including biological processes, molecular functions and cellular components. Results of KEGG pathway enrichment analysis of the intersecting genes of EC and AD. The adjusted *p*-value is implied by the color of bar; and the bubble size means the number of genes.

**Figure 6:**
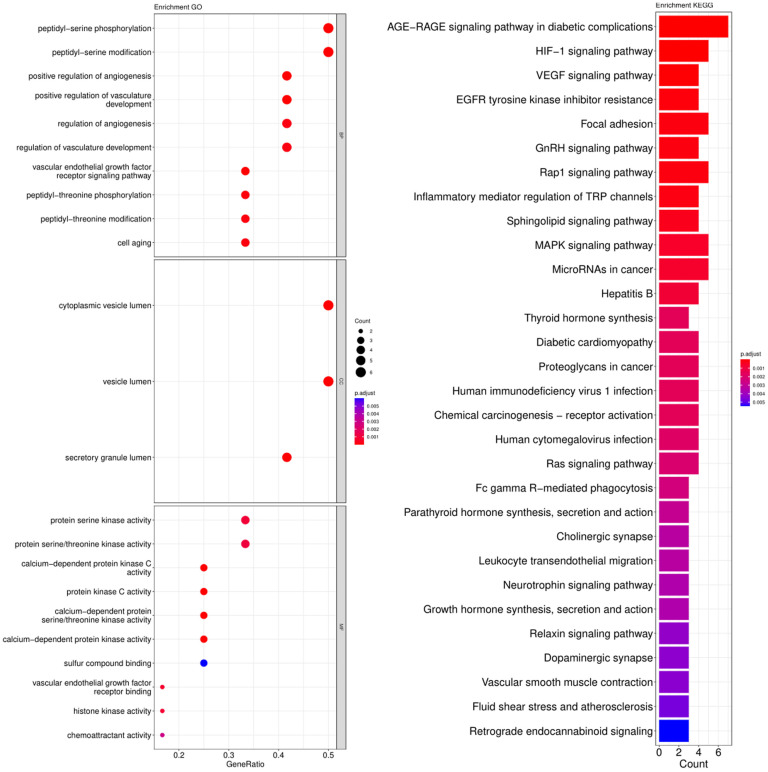
Results of GO and KEGG pathway enrichment analysis based on the common genes between ECG and AD. The visualization of GO analysis related to ECG against AD including biological processes, molecular functions and cellular components. Results of KEGG pathway enrichment analysis of the intersecting genes of ECG and AD. The adjusted *p*-value is implied by the color of bar; and the bubble size means the number of genes.

**Figure 7:**
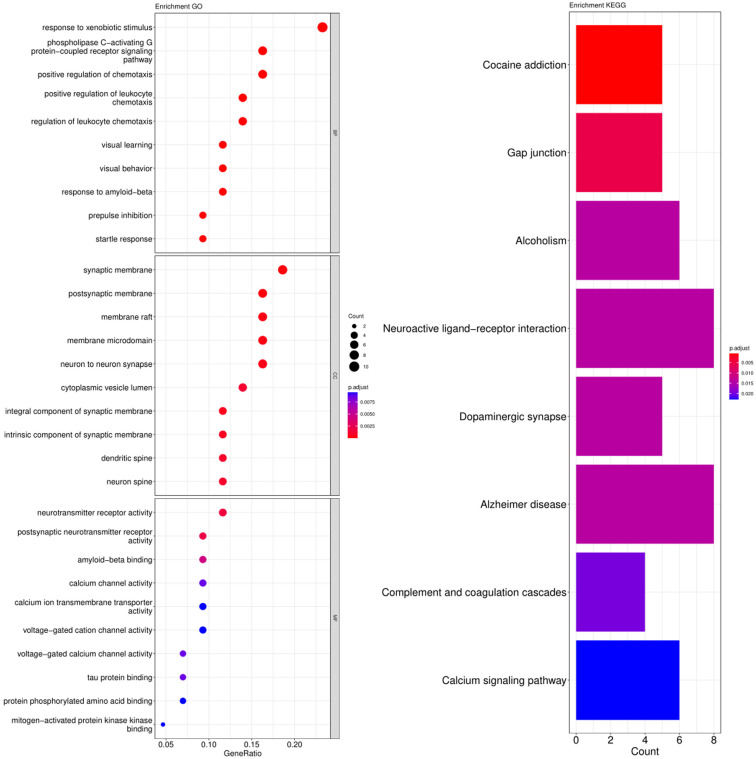
Results of GO and KEGG pathway enrichment analysis based on the common genes between EGC and AD. The visualization of GO analysis related to EGC against AD including biological processes, molecular functions and cellular components. Results of KEGG pathway enrichment analysis of the intersecting genes of EGC and AD. The adjusted *p*-value is implied by the color of bar; and the bubble size means the number of genes.

**Figure 8:**
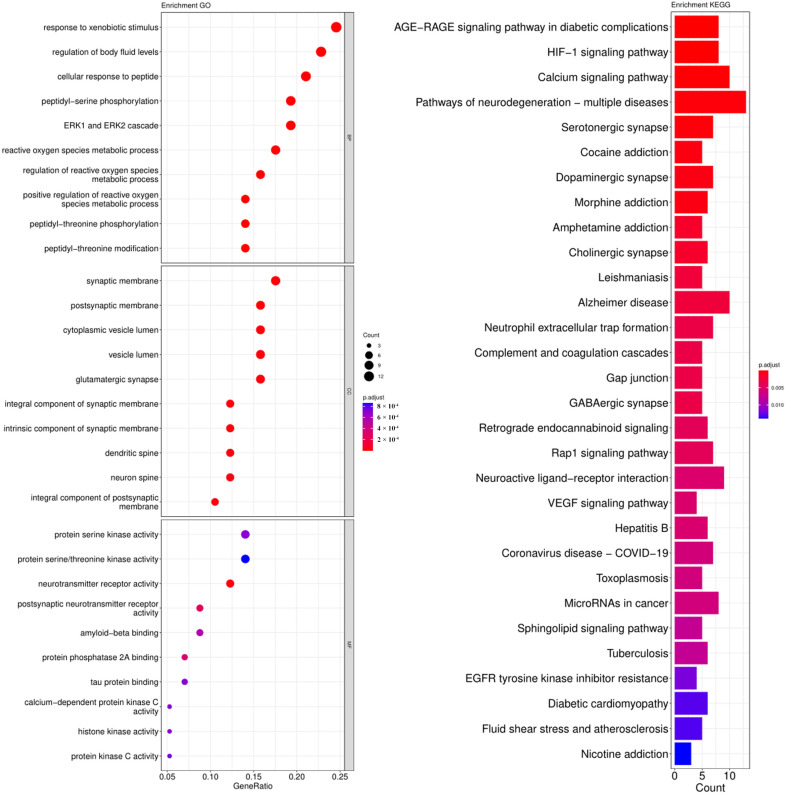
Results of GO and KEGG pathway enrichment analysis based on the common genes between EGCG and AD. The visualization of GO analysis related to EGCG against AD including biological processes, molecular functions and cellular components. Results of KEGG pathway enrichment analysis of the intersecting genes of EGCG and AD. The adjusted *p*-value is implied by the color of bar; and the bubble size means the number of genes.

The cross-genes of tea polyphenols and AD were used for the enrichment analysis of the GO and KEGG pathways. The calculated data was demonstrated as bubble charts and histograms derived from GO and KEGG (Figure 5, Figure 6, Figure 7 and Figure 8).

Response to xenobiotic stimulus, response to ethanol, regulation of chemotaxis, response to alcohol, second-messenger-mediated signaling, positive regulation of chemotaxis, positive regulation of leukocyte chemotaxis, phospholipase C-activating G protein-coupled receptor signaling pathway, regulation of leukocyte chemotaxis and positive regulation of leukocyte migration were identified as potentially relevant biological processes related to the core targets of EC (Figure 5). The parts of enriched cell components included synaptic membrane, membrane raft, postsynaptic membrane, membrane microdomain, secretory granule lumen, integral component of synaptic membrane, intrinsic component of synaptic membrane, intrinsic component of presynaptic membrane and caveola (Figure 5). Additionally, the following molecular functions were identified by GO enrichment analysis: flavin adenine dinucleotide binding, steroid binding, neurotransmitter receptor activity and others (Figure 5). The enrichment pathways identified following KEGG analysis that contained the most core targets (*p* < 0.05) included cocaine addiction, calcium signaling pathway, arginine biosynthesis, HIF-1 signaling pathway, pathways of neurodegeneration-multiple diseases, estrogen signaling pathway, chemical carcinogenesis-receptor activation, AD, complement and coagulation cascade, Gap junction and others (Figure 5).

Peptidyl-serine phosphorylation, peptidyl-serine modification, peptidyl-threonine phosphorylation, peptidyl-threonine modification, positive regulation of angiogenesis, positive regulation of vasculature development, regulation of angiogenesis, regulation of vasculature development, vascular endothelial growth factor receptor signaling pathway and cell aging were identified as biological processes related to the core targets of ECG (Figure 6). The parts of cell components that were enriched included cytoplasmic vesicle lumen, vesicle lumen and secretory granule lumen (Figure 6). Additionally, the following molecular functions were identified by GO enrichment analysis: protein serine kinase activity, protein serine/threonine kinase activity, calcium-dependent protein kinase C activity and others (Figure 6). The enrichment pathways identified following KEGG analysis that contained the most core targets (*p* < 0.05) included the AGE-RAGE signaling pathway in diabetic complications, HIF-1 signaling pathway, VEGF signaling pathway, EGFR tyrosine kinase inhibitor resistance, focal adhesion and more (Figure 6). 

Response to xenobiotic stimulus, phospholipase C-activating G protein-coupled receptor signaling, positive regulation of chemotaxis and others were identified as the biological processes related to the core targets of EGC (Figure 7). The parts of enriched cell components included synaptic membrane, postsynaptic membrane, membrane raft and more (Figure 7). Additionally, the following molecular functions were identified by GO enrichment analysis: neurotransmitter receptor activity, postsynaptic neurotransmitter receptor activity and others (Figure 7). The enrichment pathways identified following KEGG analysis that contained the most core targets (*p* < 0.05) included cocaine addiction, gap junction, alcoholism, neuroactive ligand-receptor interaction, dopaminergic synapse, AD, complement and coagulation cascades, and calcium signaling pathway (Figure 7). 

Response to xenobiotic stimulus, regulation of body fluid levels, cellular response to peptide, peptidyl-serine phosphorylation, ERK1 and ERK2 cascade and others were identified as the biological processes related to the core targets of EGCG (Figure 8). The enriched parts of cell components included the synaptic membrane, postsynaptic membrane, cytoplasmic vesicle lumen and so on (Figure 8). Additionally, the following molecular functions were identified by GO enrichment analysis: protein serine kinase activity, protein serine/threonine kinase activity, neurotransmitter receptor activity and others (Figure 8). The enrichment pathways identified following KEGG analysis that contained the most core targets (*p* < 0.05) included the AGE-RAGE signaling pathway in diabetic complications, HIF-1 signaling pathway, calcium signaling pathway, pathways of neurodegeneration-multiple diseases and more (Figure 8).

### 3.4. Quantum Chemical Calculation of Four Tea Polyphenols

**Figure 9:**
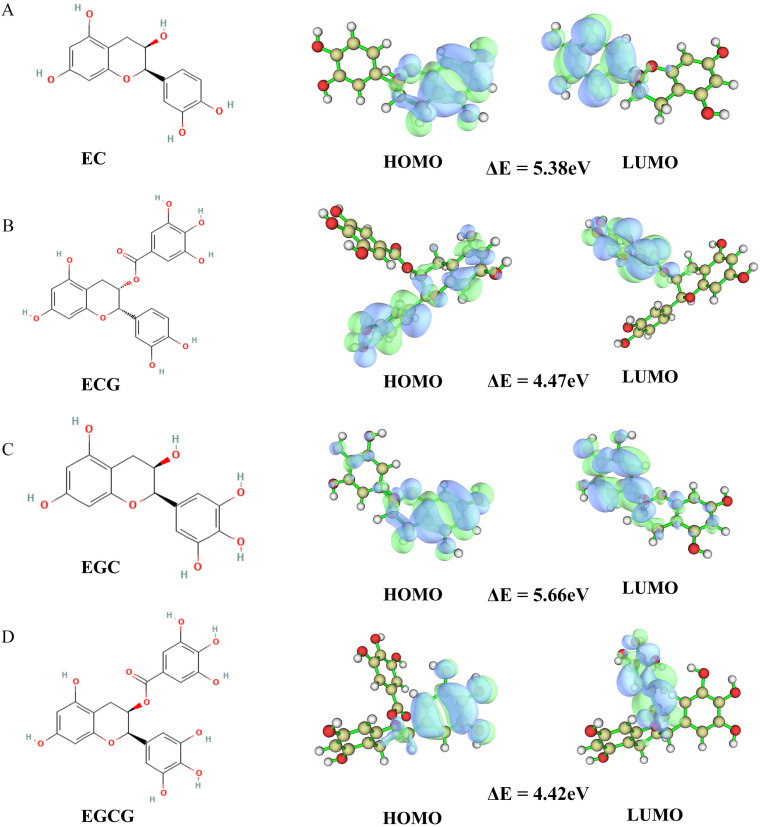
Molecular structure and HOMO–LUMO orbital diagram of four tea polyphenols. (**A**) The HOMO–LUMO orbital diagrams of EC. (**B**) The HOMO–LUMO orbital diagrams of ECG. (**C**) The HOMO–LUMO orbital diagrams of EGC. (**D**) The HOMO–LUMO orbital diagrams of EGCG.

**Figure 10:**
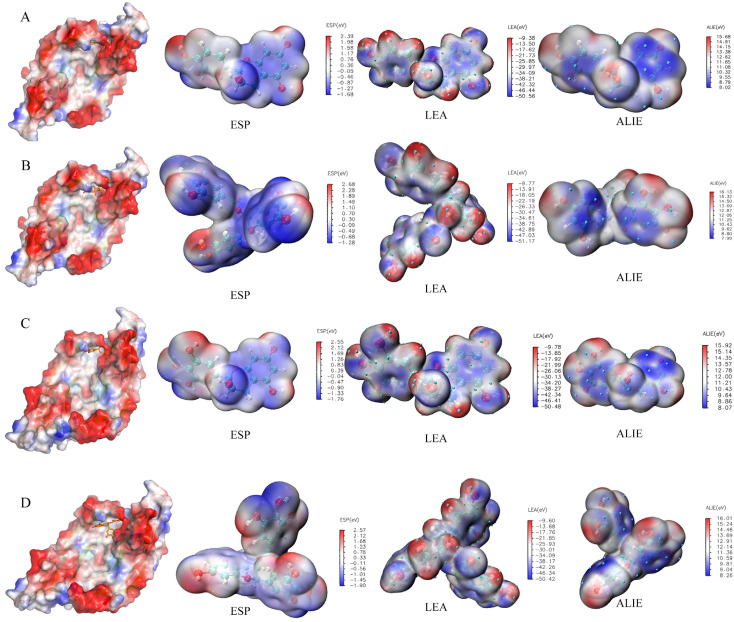
Electrostatic potential surface diagram of VEGFA, and the ESP diagrams, LEA diagrams and ALIE diagrams of the four tea polyphenols. (**A**) The ESP, LEA and ALIE diagrams of EC. (**B**) The ESP, LEA and ALIE diagrams of ECG. (**C**) The ESP, LEA and ALIE diagrams of EGC. (**D**) The ESP, LEA and ALIE diagrams of EGCG.

The B3LYP/6–31G* method was used to perform the Gaussian quantification calculation of the four tea polyphenols. First, the calculation results of the HOMO–LUMO orbit (Figure 9) were: (1) EC: HOMO orbit energy = −5.53 eV, LUMO orbit energy = −0.15 eV, and energy gap = 5.38 eV; (2) ECG: HOMO orbit energy = −5.63 eV, LUMO orbit energy = −1.16 eV, and energy gap = 4.47 eV; (3) EGC: HOMO orbit energy = −5.58 eV, LUMO orbit energy = 0.08 eV, and energy gap = 5.66 eV; and (4) EGCG HOMO orbit energy = −5.47 eV, LUMO orbit energy = −1.05 eV, and energy gap = 4.42 eV. Next, the results of the electrostatic potential, local electron affinity energy and average local ionization energy diagram (Figure 10) of the Gauss calculation indicated that the four tea polyphenols molecules were closely bound to VEGFA.

### 3.5. Binding of Four Tea Polyphenols to VEGFA

**Figure 11:**
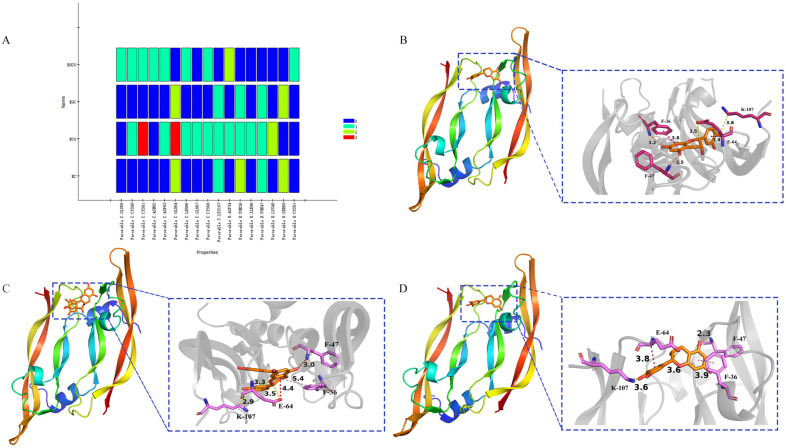
Complex fingerprint analysis of EC, ECG, EGC and EGCG (**A**) and molecular docking indicated the possible binding sites between AD and EC (**B**), ECG (**C**), and EGC (**D**). For EC, ECG and EGC, the key residues for target genes (VEGFA) may be GLU-64, LYS-107, PHE-36 and PHE-47.

The pre-docking protein of VEGFA, the intersection protein of the top 10 key proteins between four tea polyphenols and AD, was identified using the Uniprot and protein data bank databases. Then, the compound structure with PDB ID 4QAF was determined and used for molecular docking with the four tea polyphenols. Since VEGFA had no effective small-molecule-binding crystal structure, we estimated the binding site of the tea polyphenols and VEGFA according to the polypeptide’s binding site. Using the information on active sites in the structure of 4QAF protein, we determined the active sites based on the positions of C: TYR25, D: TYR45, ARG82, ILE83, PRO85, HIS86, GLY88, GLN89 and HIS90. In VEGFA (4QAF), we set the input site sphere parameter to 2.27147, 58.0352, 9.31752 and 15.027 (the coordinates and radius of the sphere), accompanied by 1.80 Å RMSD in the original protein. The conventional hydrogen bonds between EC and amino acid residues were PHE36, SER50 and PHE47, and the carbon-hydrogen bonds between them were LYS107 and GLU64. The conventional hydrogen bonds between ECG and amino acid residues were PHE47, LYS48, LEU66, ASP63 and CYS68, and the carbon-hydrogen bonds between them were LYS107, GLU64, GLU67 and ASP34. The conventional hydrogen bonds between EGC and amino acid residues were SER50 and PHE47, and the carbon-hydrogen bonds between them were LYS107 and GLU64. The conventional hydrogen bonds between EGCG and amino acid residues were CYS68, GLY59, CYS61, ASN62 and ASP63, and the carbon-hydrogen bonds between them were ASP34 and LEU66. According to the compound fingerprint analysis, the common residues of the three tea polyphenols EC, ECG and EGC were GLU64, LYS107, PHE36 and PHE47 (Figure 11). Overall, these findings suggested that these residues could be the potential key residues of green tea acting on AD, while EGCG might be dissimilar to the other three tea polyphenols due to structural problems.

### 3.6. Alanine Scanning of the Binding Sites

After alanine scanning, we discovered that the key residues of EC for VEGFA might be GLU64 and PHE36 (Table 1), the key residues of ECG for target protein were GLU64, PHE36, CYS61 and LYS48 (Table 2), the key residues of EGC for target protein were GLU64, PHE36 and SER50 (Table 3), and the key residues of EGCG for target protein were GLU64 and LYS48 (Table 4).

## 4. Discussion

In this study, network pharmacological analysis was conducted to reveal the biological processes, pathways and mechanisms of four tea polyphenol active molecules against AD [55]. The bioinformatics research results revealed that the core intersection target was VEGFA. Molecular docking analysis and quantitative analysis of four kinds of tea polyphenols revealed the binding activity of the four kinds of tea polyphenols with VEGFA structure in AD, indicating that EC, ECG and EGC could effectively bind to specific proteins in patients with AD, as well as the key residues of interaction by combining alanine scanning. VEGFA has important functions, such as directly regulating the various ion channels on neuronal cell membranes, the transmission of excitatory and inhibitory neurotransmitters, and the development and regeneration of neurons. The neuroprotective effects of VEGFA include reducing the degree of ischemic brain injury, reducing the death of nerve cells in the damaged brain, narrowing the foci of cerebral infarction, etc. In addition, in the case of brain injury, VEGFA promotes the transformation of glial cells into neurons, and functions as an important regulatory protein in the structural and functional remodeling of neurovascular units [56,57]. For the selected key target gene VEGFA, on the one hand, relevant experiments have proved that decreased levels of VEGFA in serum and cerebrospinal fluid are linked with increased risk for AD and cognitive impairments [58,59], while on the other hand, it has been reported that EGCG could inhibit wild-type EGFR as well as some of its mutated forms [60]. Moreover, it has been shown that green tea, black tea and oolong tea polyphenols could increase the gene expression of VEGFA [61].

We investigated the potential role of four tea polyphenols in the treatment of AD by assessing their effects on different biological processes and molecular functions related to AD. GO bio-processes of the four tea polyphenols emphasized the response to xenobiotic stimuli, including ADAM17, MDK, TP53, STAT1 and more. ADAM17 is a transmembrane metalloproteinase involved in the shedding of extracellular domains of various proteins, and has the characteristics of adhesion and proteolysis. TNF-α plays a key role in processing many other substrates, such as cell adhesion molecules, cytokines, growth factor receptors and epidermal growth factor receptors (EGFR), and in activating the Notch signaling pathway [62]. γ-Secretase is an important cleaving enzyme that activates the Notch pathway. It is also a cleaving enzyme of amyloid precursor protein (APP) related to AD. Midkine (MK or MDK) is a 15 kDa heparin-binding molecule known as neurite growth-promoting factor-2 (NEGF2). The nervous system is an essential part of the human body, and the repair and regeneration of injured axons are of great significance for maintaining the function of the nervous system, and are closely related to neurodegenerative diseases. The regenerative ability of the peripheral nervous system (PNS) differs from that of the central nervous system (CNS). The axons of the peripheral nervous system can often regenerate themselves after being damaged to a certain extent, while the axons of the central nervous system are difficult to recover after damage. The p53 protein is directly involved in the formation of AD. The transcription factor STAT1 regulates the type I interferon signaling pathway in SCD, and the down-regulation of interferon signaling activity might increase the risk of AD progression [63]. Here, our data showed that tea polyphenols might be an alternative treatment for AD. In regard to molecular functions, we discovered that tea polyphenols regulated the activities of protein serine kinase, neuron transmitter–receivers and others in AD.

Our results also indicated that the four tea polyphenols could exert their anti-AD effects through the calcium signaling pathway, which is one of the main signaling pathways in neurodegenerative diseases. It had previously been confirmed that an increase in calcium ion and cAMP levels promoted axon regeneration [64]. Additionally, it had been reported that green tea polyphenols facilitated Ca2+-dependent glutamate release [65]. Apart from calcium signals, the results of our KEGG analysis indicated the potential implications of many nerve-related signals, including the HIF-1 signaling pathway, AGE–RAGE signaling pathway in diabetic complications, MAPK signaling pathway and neurodegenerative disease pathway. The expression of HIF-1 could induce the accumulation of the p53 protein [66], which participates in the formation of AD. A previous study also showed that a prodrug of EGCG (pro-EGCG) alleviated mouse laser-induced choroidal neovascularization (CNV) leakage and reduced CNV area by down-regulating the HIF-1α/VEGF/VEGFR2 pathway [67]. Green tea polyphenols can down-regulate caveolin-1 expression via ERK1/2 and p38MAPK in endothelial cells [68]. Advanced glycation end products (AGEs)-receptor of advanced glycation end products (RAGE) signaling pathway is an important link in the occurrence and development of diabetic nephropathy, whose mechanism of action is mainly to activate the transcription of nuclear factor-κB (NF-κB) and stimulate the production of vascular endothelial growth factor (VEGF) [69], proving that VEGFA is a key target protein for the interaction between AD and tea polyphenols.

Overall, this study revealed that the four tea polyphenols mainly showed their anti-AD mitigation effects through responses to xenobiotic stimulus. In addition, bioinformatics analysis showed that the investigated tea polyphenols affected AD via calcium channels, HIF-1 channels and others. Lastly, our analyses also revealed the promising role of VEGFA as a potential biomarker for diagnosing AD.

## 5. Conclusions

By assessing the enrichment of intersection genes in AD, we revealed that tea polyphenols exerted their anti-AD effects via the calcium signaling and neurodegenerative disease signaling pathways. VEGFA was the common key target protein of the four tea polyphenol molecules, and Glu64 and Phe36 were identified as the key residues of interaction. In conclusion, using network pharmacological analysis, we comprehensively revealed the biological processes, targets and molecular mechanisms of EC, EGC, ECG and EGCG in AD, and showed that they could serve as promising compounds for treating AD.

## Figures and Tables

**Table 1:** Alanine scanning results of EC docking sites.

Index	Mutation	Mutation Energy (kcal/mol)	Effect
1	S50A	−0.23	NEUTRAL
2	C61A	−0.01	NEUTRAL
3	E67A	0.02	NEUTRAL
4	D63A	0.03	NEUTRAL
5	L66A	0.05	NEUTRAL
6	I35A	0.23	NEUTRAL
7	F47A	0.36	NEUTRAL
8	D34A	0.41	NEUTRAL
9	E64A	0.61	DESTABILIZING
10	F36A	1.2	DESTABILIZING

**Table 2:** Alanine scanning results of ECG docking sites.

Index	Mutation	Mutation Energy (kcal/mol)	Effect
1	E67A	0	NEUTRAL
2	G59A	0.01	NEUTRAL
3	C68A	0.01	NEUTRAL
4	D63A	0.03	NEUTRAL
5	L66A	0.12	NEUTRAL
6	S50A	0.36	NEUTRAL
7	C61A	0.55	DESTABILIZING
8	F36A	0.64	DESTABILIZING
9	K48A	0.78	DESTABILIZING
10	E64A	0.79	DESTABILIZING

**Table 3:** Alanine scanning results of EGC docking sites.

Index	Mutation	Mutation Energy (kcal/mol)	Effect
1	S50A	−0.58	STABILIZING
2	C61A	−0.19	NEUTRAL
3	D63A	−0.06	NEUTRAL
4	N62A	−0.04	NEUTRAL
5	I46A	0	NEUTRAL
6	E67A	0.18	NEUTRAL
7	F47A	0.28	NEUTRAL
8	D34A	0.37	NEUTRAL
9	E64A	0.53	DESTABILIZING
10	F36A	0.98	DESTABILIZING

**Table 4:** Alanine scanning results of EGCG docking sites.

Index	Mutation	Mutation Energy (kcal/mol)	Effect
1	C61A	−0.32	NEUTRAL
2	L66A	−0.12	NEUTRAL
3	G59A	−0.08	NEUTRAL
4	F47A	−0.04	NEUTRAL
5	D63A	0.02	NEUTRAL
6	I46A	0.36	NEUTRAL
7	S50A	0.45	NEUTRAL
8	D34A	0.46	NEUTRAL
9	E64A	0.61	DESTABILIZING
10	K48A	0.84	DESTABILIZING

## Data Availability

The data are available from the corresponding author.
